# Vitiligo and Autoimmune Thyroid Disorders

**DOI:** 10.3389/fendo.2017.00290

**Published:** 2017-10-27

**Authors:** Enke Baldini, Teresa Odorisio, Salvatore Sorrenti, Antonio Catania, Francesco Tartaglia, Giovanni Carbotta, Daniele Pironi, Roberta Rendina, Eleonora D’Armiento, Severino Persechino, Salvatore Ulisse

**Affiliations:** ^1^Department of Surgical Sciences, “Sapienza” University of Rome, Rome, Italy; ^2^Laboratory of Molecular and Cell Biology, Istituto Dermopatico dell’Immacolata-IRCCS, Rome, Italy; ^3^Department of Internal Medicine and Medical Specialties, “Sapienza” University of Rome, Rome, Italy; ^4^NESMOS Department, “Sapienza” University of Rome, Rome, Italy

**Keywords:** vitiligo, autoimmune thyroid diseases, tyrosinase, TSH receptor, thyroglobulin, reactive oxygen species, CD8^+^ T cells, autoimmune polyendocrine syndromes

## Abstract

Vitiligo represents the most common cause of acquired skin, hair, and oral depigmentation, affecting 0.5–1% of the population worldwide. It is clinically characterized by the appearance of disfiguring circumscribed skin macules following melanocyte destruction by autoreactive cytotoxic T lymphocytes. Patients affected by vitiligo usually show a poorer quality of life and are more likely to suffer from depressive symptoms, particularly evident in dark-skinned individuals. Although vitiligo is a non-fatal disease, exposure of affected skin to UV light increases the chance of skin irritation and predisposes to skin cancer. In addition, vitiligo has been associated with other rare systemic disorders due to the presence of melanocytes in other body districts, such as in eyes, auditory, nervous, and cardiac tissues, where melanocytes are thought to have roles different from that played in the skin. Several pathogenetic models have been proposed to explain vitiligo onset and progression, but clinical and experimental findings point mainly to the autoimmune hypothesis as the most qualified one. In this context, it is of relevance the strong association of vitiligo with other autoimmune diseases, in particular with autoimmune thyroid disorders, such as Hashimoto thyroiditis and Graves’ disease. In this review, after a brief overview of vitiligo and its pathogenesis, we will describe the clinical association between vitiligo and autoimmune thyroid disorders and discuss the possible underlying molecular mechanism(s).

## Vitiligo: An Overview

Vitiligo represents the most common cause of acquired skin, hair and oral depigmentation, and often occurs as an inherited disease (1). Clinically, it is characterized by the progressive loss of melanocytes causing the appearance of well-circumscribed milky/white cutaneous macules. Histologically, skin lesions show basal hypopigmentation and increased dermal inflammation relative to perilesional normal skin, with complete or near-complete loss of melanocytes at the basal epidermal layer (2). Following the Vitiligo Global Issues Consensus Conference in 2011, the disease has been categorized based on clinical parameters into: segmental vitiligo (SV), non-segmental vitiligo (NSV), and mixed vitiligo (MV) (1). SV is characterized by a unilateral distribution of the macules and is less common compared with the NSV, which shows symmetrical and bilateral white patches (3). NSV includes different clinical vitiligo subtypes, namely, acrofacial, generalized, mucosal, and universal vitiligo. NSV may be initially classified as acrofacial and, over time, be reclassified as generalized or universal vitiligo. On the other hand, MV includes the combination of an initial SV followed by the occurrence, after several months or years, of bilateral NSV patches (1, 4).

The prevalence of vitiligo has been estimated to be 0.5–1% of the world population. However, it can vary from country to country. In fact, the prevalence recorded in Denmark is 0.38%, whereas in India it is up to 8.8% (1, 5, 6). Vitiligo can arise at any age, even if about 50% of cases are diagnosed before the age of 20, and both sexes are equally affected (1, 6).

Due to its disfiguring effects, vitiligo may have a detrimental impact on patient’s quality of life (QoL) and mental health (7–9). A recent review of studies published over the last two decades indicates that women show more QoL impairment than men, married women more than singles, young patients more than elderly ones, and dark-skinned people more than white people (7, 8). Moreover, a recent meta-analysis demonstrated that vitiligo patients were significantly more likely to suffer from depression (9). Although vitiligo is a non-fatal disease, exposure of affected skin to UV light increases the chance of skin irritation and cancer (10). Furthermore, vitiligo has been associated with other rare systemic disorders, including the Vogt–Koyanagi–Harada, the Kabuki, and mitochondrial encephalopathy, lactic acidosis, and stroke-like episodes (MELAS) syndromes due to the presence of melanocytes in other parts of the body, such as in eyes, auditory, nervous, and cardiac tissues, where melanocytes are thought to have different roles from that played in the skin (11). In particular, the Vogt–Koyanagi–Harada disease is an autoimmune multisystemic disorder branded by granulomatous panuveitis with exudative retinal detachments, neurologic and hearing manifestations, and vitiligo. The Kabuki syndrome manifests with abnormalities in multiple organ systems and is characterized by distinctive facial features, including arched eyebrows, long eyelashes, long openings of the eyelids with the everted lower lids, and large protruding earlobes. It usually associates with autoimmune diseases such as idiopathic thrombocytopenic purpura, hemolytic anemia, thyroiditis, and vitiligo. The MELAS syndrome is a mitochondrial disorder due to mutations of the mitochondrial genome. The typical presentation of patients with MELAS syndrome is described by the name of the disorder. Additional features are seizures, diabetes mellitus, hearing loss, cardiac disease, short stature, endocrinopathies, neuropsychiatric dysfunctions and skin alterations including hypertrichosis, eczema, and vitiligo.

## Pathogenesis

Although several hypotheses have been put forward to explain vitiligo ethiopathogenesis, the autoimmune theory is the most accredited one, being sustained by several epidemiological, clinical, and experimental findings (4, 9, 11–17). These studies indicate that melanocyte defects drive vitiligo pathogenesis by triggering, in susceptible individuals, an autoimmune response that leads to melanocyte destruction (11). Several exogenous and endogenous stimuli have been linked to the onset of the disease. The exogenous factors include ultraviolet irradiations, trauma (Koebner phenomenon), stress, major infections, malignancies, neural abnormalities, vaccinations, pregnancy, calcium imbalance, certain drugs, hormones, and exposure to cytotoxic compounds. Among the endogenous factors are melanin synthesis, cellular metabolism, proliferation, differentiation, apoptosis, and immune reactions (11, 14, 18–20). All of these are thought to induce oxidative stress in melanocytes, as indicated by the high levels of reactive oxygen species (ROS), mainly hydrogen peroxide and peroxinitrite, found in lesional skin (11, 14, 18–20). The ROS increase may also result from compromised antioxidant responses with local and/or systemic imbalance of the antioxidant systems (11, 14, 18–20). For example, the superoxide dismutase is present at higher levels in perilesional skin and patient’s sera (17, 20), whereas the level of the antioxidant enzyme catalase was found reduced in the vitiliginous skin compared with normal skin (20). The important role played by the antioxidant system in the pathogenesis of vitiligo is further corroborated by a recent study showing the association between a single nucleotide polymorphism of the nuclear factor, erythroid 2 like 2 (*NRF2*) gene and vitiligo (19, 21). The transcription factor Nrf2 regulates genes containing the antioxidant response elements (AREs) in their promoters and encoding proteins that protect against oxidative damage triggered by injury and inflammation. In addition, it has been shown that Nfr2-ARE/heme oxygenase-1 pathway is functionally deficient in the disease-free epidermis of patients with vitiligo (22). This is in agreement with very recent findings showing the ability of simvastatin to protect human melanocytes from H_2_O_2_-induced oxidative stress by activating Nrf2 (23). Finally, reduced levels of non-enzymatic antioxidants such as beta-carotene, ubiquinone, vitamins E and C, ferritin, and metallothionein may contribute to the increased amount of ROS observed in vitiliginous melanocytes (19).

Oxidative stress may affect the structure and functions of the endoplasmic reticulum (ER), which act as a cellular stress sensor. Dilation of the ER is a hallmark of melanocytes at the periphery of vitiligo lesions, and the disruption of redox reactions, critical for proper protein folding, causes the accumulation of immature proteins and misfolded peptides leading to the activation of the unfolded protein response (UPR) (24, 25). The latter, under sustained cellular stress, promotes autoimmune responses *via* apoptotic cascades (19). Actually, exposure to chemical triggers of vitiligo was shown to induce oxidative stress and to promote UPR activation in melanocytes (26). The importance of the UPR in the pathogenesis of vitiligo is further corroborated by several lines of experimental evidence, which identified the X-box binding protein 1 (*XBP1*) gene, encoding a transcription factor mediating UPR activation, as a susceptibility locus for generalized vitiligo (27–30). The UPR induces also the expression of cytokines, such as IL-6, IL-8, IL-11, and tumor necrosis factor, and can attract cells of the innate immune system to the skin of vitiligo patients, as documented by the aberrant activation of natural killer and dendritic cells (DCs) in lesional skin (11). More recently, a role for calreticulin (CRT), an ER protein regulating intracellular Ca^2+^, has been proposed in the progression of vitiligo (19). In particular, a redistribution of CRT from the ER lumen to the plasma membrane of melanocytes takes place under oxidative stress (19). Surface CRT is thought to direct the contact of stressed melanocytes with DCs, eliciting downstream immune responses and melanocyte apoptosis. The latter provides abundant antigenic peptides to the antigen-presenting cells leading to the activation of T cells, thus promoting autoimmunity. In this context, it is also worth to consider that the increased ROS levels are thought to modify tyrosinase (TYR) and other melanogenic proteins into neoantigens (11). Indeed, patients affected by vitiligo show circulating autoantibodies directed toward specific melanocyte antigens such as TYR, tyrosinase-related protein-1 (TRP-1), TRP-2, Pmel17 (or gp100), and type 1 membrane receptor for melanin-concentrating hormone, whose serum level correlates with the disease severity (11, 31–36). In early lesions, CD8^+^ cytotoxic T lymphocytes have been found close to melanocytes, and a perivascular lymphocytic infiltrate could be appreciated at the expanding edge of active skin lesions (37). In addition, the concentration of melanocyte-specific CD8^+^ T cells is higher in the blood of patients affected by vitiligo and correlates with disease activity (11, 31, 38). Furthermore, interferon-γ (IFN-γ) has been shown to play a central role in vitiligo progression through the release of several chemokines, such as CXCL9, 10, and 11 (17, 39). It has been also suggested that IFN-γ could play a direct role in vitiligo pathogenesis following the observation that the IFN-γ derived from cytotoxic T cells could itself cause apoptosis in melanocytes (40). This is in agreement with recent studies showing that human vitiligo as well as a mouse model of vitiligo reflects an IFN-γ-specific Th1 immune response in the skin that involves IFN-γ-dependent chemokines (41–44).

Recent findings indicate the participation in this process of TH17 cells, identified in the lesional skin of vitiligo patients (45, 46). The TH17 cells, by releasing interleukin-17, may induce in activated immune cells secretion of proinflammatory cytokines, which in turn recruit and activate mononuclear lymphocytes, strongly involved in disease progression (46). Finally, regulatory T cells (Treg), which are in charge to maintain peripheral tolerance through the suppression of self-reactive T cells, appear reduced in number and functionally flawed in lesional skin of patients affected by vitiligo (47).

A number of studies have shown that the uptake by keratinocytes of the melanocyte released melanosomes take place through phagocytic ingestion in a receptor-mediated process, involving the protease-activated receptor-2 and keratinocyte growth factor receptor/fibroblast growth factor receptor 2b (KGFR/FGFR2b) (48–50). A recent work reported a decreased expression of KGF/FGF7 and its receptor in pathological hypopigmented skin, which may contribute to the formation of the classical milky macules of vitiligo (50).

Finally, it is worth to mention that a number of genome-wide association and genetic linkage studies identified more than 30 different genes related to an increased risk of vitiligo, the majority of which are immune genes implicated in both the innate and the adaptive immune responses (4, 9, 11, 13).

## Association with Autoimmune Thyroid Diseases (AITD)

Besides the abovementioned involvement of the immune system in vitiligo pathogenesis, epidemiological evidence further corroborates the autoimmune genesis of vitiligo. In particular, vitiligo is present within the autoimmune polyendocrine syndromes (51), and it is more frequently encountered in family members of patients affected by autoimmune diseases, such as inflammatory bowel disease, psoriasis, rheumatoid arthritis, type 1 diabetes, systemic lupus erythematosus, pernicious anemia, and AITD (31, 52–69). The latter, as outlined in several studies performed over the last decades, represent the most frequent autoimmune disorders associated with vitiligo (54, 58, 66–73). A recent meta-analysis, performed on 48 articles published between 1968 and 2012, showed that in patients affected by vitiligo the prevalence of AITD was 14.3%, while positivity to thyroid-specific antibodies [i.e., anti-thyroglobulin (Tg), anti-thyroid peroxidase, and anti-thyrotropin receptor (TSHR)] was found in 20.8% of them (74). Moreover, the presence of anti-thyroid hormones antibodies in the serum of patients affected by vitiligo was detected in 77 out of 79 vitiligo patients analyzed, suggesting a possible pathogenetic role (70, 75). *Vice versa*, the prevalence of vitiligo among AITD patients has been reported to vary from 2.7 to 7% (66, 67, 76, 77). It is also worth to note that the risk of thyroid disease in vitiligo patients increases with age (71, 74). All together, these findings have led to the recommendation of screening patients affected by vitiligo for thyroid diseases and thyroid autoantibodies, in an effort to detect undiagnosed thyroid diseases or to assess the risk of future onset (74, 78).

## Molecular Mechanisms Underlying Vitiligo and Thyroid Autoimmune Disease Association

The reported association of vitiligo with AITD suggests the presence of shared heritable susceptibility genes (79–87). Thirty-seven susceptibility genes have been identified for vitiligo disease and more than 15 for AITD (79–87). Genome-wide linkage analysis and candidate gene association studies identified nine loci potentially involved in both AITD and vitiligo (79–81). Among these, there are organ-specific genes such as those coding for TYR, Tg, and TSHR (81–85). In addition, an autoimmunity susceptibility locus (AIS1) was identified by genome-wide linkage analysis on chromosome 1 in families characterized by vitiligo and Hashimoto’s thyroiditis (HT) (86–88). Among the 27 genes mapping to the AIS1 locus, the forkhead transcription factor D3 appears to be the most plausible responsible for the concomitant occurrence of vitiligo and AITD (86, 89). In addition, a single nucleotide polymorphism of the *PTPN22* gene, encoding a lymphoid specific phosphatase, is shared among patients with vitiligo and AITD (79). These findings suggest that the association observed between vitiligo and AITD could be explained, at least in part, by the sharing of a subset of susceptibility genes.

Of interest are the recently reported observations showing melanocyte-specific antigen expression in thyroid tissues of patients with HT, as well as in thyroid tissues of healthy individuals (88). In particular, thyroid tissues from HT patients without vitiligo, and normal thyroid tissues, were both negative for the expression of NKI/beteb, Pmel17, TRP-1, HMB-45, and S100, whereas they were positive for the expression of TRP-2, lysosome-associated membrane protein 1 (LAMP1), and CD69. Interestingly, TYR was only detected in thyroid from HT patients. Moreover, levels of LAMP1 and CD69 were higher in thyroid with HT compared with normal thyroid (90). The differences in type and amount of melanocyte antigens observed in the thyroid of HT patients may provide the immunological basis for secondary vitiligo associated with HT. *Vice versa*, different skin cell types, including keratinocytes, dermal fibroblasts, and melanocytes, have been shown to express functional TSHR and other thyroid-specific antigens including Tg, thyroperoxidase, and natrium/iodide symporter (91, 92). Thus, it may be speculated that in vitiligo patients the activation of the immune system against these antigens expressed in vitiliginous melanocytes may cause a secondary AITD.

## Conclusion

Knowledge regarding the pathogenesis of vitiligo has considerably increased over the last decades starting to clarify the molecular mechanisms underlying disease etiology and progression, as well as the association with other autoimmune disorders. Several susceptibility genes have been identified in both vitiligo and AITD patients that, along with the identification of shared antigens between melanocytes and thyrocytes, may contribute to explain the observed association between AITD and vitiligo.

## Author Contributions

All the authors contributed to the first draft of the article and its revision and approved its final version.

## Conflict of Interest Statement

The authors declare that the research was conducted in the absence of any commercial or financial relationships that could be construed as a potential conflict of interest. The reviewers SF and IR and handling editor declared their shared affiliation.
